# Subject satisfaction and psychological well‐being with escalating abobotulinumtoxinA injection dose for the treatment of moderate to severe glabellar lines

**DOI:** 10.1111/jocd.14906

**Published:** 2022-04-12

**Authors:** Steven Dayan, John Joseph, Amir Moradi, Z. Paul Lorenc, Kyle Coleman, Glynis Ablon, Joely Kaufman‐Janette, Sue Ellen Cox, Andrew Campbell, Girish Munavalli, Inna Prygova

**Affiliations:** ^1^ DeNova Research Chicago Illinois USA; ^2^ Clinical Testing of Beverly Hills Encino California USA; ^3^ Moradi M.D. Vista California USA; ^4^ Lorenc Aesthetic Plastic Surgery Center New York USA; ^5^ Etre Cosmetic Dermatology and Laser Center New Orleans Louisina USA; ^6^ Ablon Skin Institute and Research Center Manhattan Beach California USA; ^7^ Skin Research Institute, LLC, Coral Gables Florida USA; ^8^ Aesthetic Solutions, PA Chapel Hill North Carolina USA; ^9^ Quintessa Aesthetic Center – EthiQ2 Research, LLC Mequon Wisconsin USA; ^10^ Dermatology, Laser & Vein Specialists of the Carolinas PLLC Charlotte North Carolina USA; ^11^ Galderma Uppsala Sweden

**Keywords:** abobotulinumtoxinA, botulinum toxin type A, FACE‐Q, global aesthetic improvement scale, moderate to severe glabellar lines, psychological well‐being, satisfaction

## Abstract

**Background:**

Previous studies indicate that the efficacy and durability of a single AbobotulinumtoxinA (ABO) treatment for moderate to severe glabellar lines may be enhanced with increasing dose, while safety outcomes remain consistent with those of the licensed dose (50 U).

**Aims:**

Evaluation of subject‐reported indicators of treatment efficacy, satisfaction, and psychological well‐being with ABO dose escalation.

**Methods:**

A Phase 2, 36‐week, multicenter, randomized, dose‐ranging, double‐blind, placebo‐controlled study was conducted in adults with moderate to severe glabellar lines. Subjects received a single ABO treatment, dosed at 50, 75, 100, or 125 U, or placebo. Efficacy endpoints comprised subject‐assessed improvement in line severity of ≥1‐grade from baseline at maximum frown, global aesthetic improvement scale (GAIS) grade, FACE‐Q™ appraisal of lines, psychological well‐being and age, and subject satisfaction.

**Results:**

The study included 399 subjects (88.2% were female). Respective responder rates (≥1‐grade improvement) with ABO 50–125 U doses ranged between 96.3%–100% at Week 4, 65.0%–67.9% at Week 24, and 33.8%–44.4% at Week 36. GAIS responder rate and FACE‐Q appraisal of lines showed a similar pattern of change. Satisfaction was high and psychological well‐being was improved from Week 4 through Week 36, with natural, youthful, and refreshed appearance reported for all ABO doses.

**Conclusions:**

A single ABO treatment (dosed at 50–125 U) provided significant and sustained improvements in glabellar line severity over durations up to 36 weeks, versus placebo. Treatment satisfaction was high with all doses. Participants reported natural and youthful appearance, alongside improvements in psychological well‐being.

## 1 INTRODUCTION

Botulinum toxin type A treatment is the most widely used nonsurgical aesthetic facial procedure.^1^ AbobotulinumtoxinA (ABO), available as Dysport^®^ in the United States and Azzalure^®^ in Europe (Ipsen Biopharm Limited), has demonstrated efficacy, safety, and subject satisfaction in the correction of moderate to severe glabellar lines and beneficial psychological outcomes are reported post‐treatment.^2^, ^3^, ^4^, ^5^, ^6^, ^7^, ^8^, ^9^, ^10^, ^11^, ^12^, ^13^ Satisfaction with aesthetic outcome is an important indicator of treatment success, with individuals achieving ≥1‐grade improvement in glabellar line severity typically reporting high levels of satisfaction following ABO treatment.^6^, ^7^, ^11^, ^12^, ^13^ Recipients experience sustained improvements in self‐confidence and feelings of attractiveness.^12^, ^13^ Psychological benefits associated with aesthetic treatment may enhance quality of life (QoL) and motivate individuals to seek further repeat treatments.^14^, ^15^, ^16^ Specialist tools, such as the FACE‐Q™ instrument, provide a standardized model to capture satisfaction with appearance and health‐related QoL following aesthetic procedures.^17^, ^18^, ^19^ Over recent years, subject satisfaction and psychological well‐being parameters have been increasingly incorporated within randomized and observational studies examining the aesthetic efficacy of botulinum toxin type A treatments for facial lines, reflecting the impact of such treatments on QoL and their importance as a measure of effectiveness.^2^, ^5^, ^6^, ^7^, ^10^, ^12^, ^13^, ^14^, ^15^, ^16^, ^17^

Treatment efficacy and safety outcomes are well‐established with the licensed ABO 50 Speywood unit (50 U) dose.^3^, ^4^, ^5^, ^6^, ^7^, ^8^, ^9^, ^10^, ^11^ A single treatment provides significant improvements in glabellar line severity, typically lasting 4–5 months.^3^, ^4^, ^5^, ^6^, ^7^, ^8^, ^9^, ^10^, ^11^ However, emerging data indicate that higher ABO doses may prolong aesthetic effect for up to 6–9 months without impacting safety or tolerability.^5^, ^20^, ^21^ The present study was a Phase 2, 9‐month, multicenter, randomized, dose‐ranging, double‐blind, placebo‐controlled study in which individuals with moderate to severe glabellar lines received a single treatment at doses ranging between 50 and 125 U. The main results of the study, reported by Joseph et al, revealed a tendency toward higher response rates, with subjects achieving ≥2‐grade improvements alongside severity scores of 0 (none) or 1 (mild), and longer duration of aesthetic effect with increasing ABO dosage when assessed over a 9‐month period.^21^ Incidence of adverse events was consistently low across all ABO doses and comparable with the literature regarding safety with the licensed dose.^3^, ^4^, ^5^, ^6^, ^7^, ^8^, ^9^, ^10^, ^11^, ^12^, ^13^, ^21^ Here, we report subject self‐assessment of efficacy, as well as satisfaction and FACE‐Q data from the same ABO dose‐ranging study with the aim of advancing understanding regarding the relationship between treatment efficacy, satisfaction, and psychological well‐being with escalating ABO dose.^21^ Given the acknowledged relationship between successful aesthetic procedures and improvements in psychological well‐being, it is important to understand how extended ABO efficacy may influence key subject‐reported outcomes including treatment satisfaction, self‐esteem, and QoL.^5^, ^12^, ^13^, ^14^, ^15^, ^16^

## 2 METHODS

### 2.1 Study design, population, and treatment

A 9‐month, Phase 2, multicenter, randomized, dose‐ranging, double‐blind, placebo‐controlled study was conducted at 15 study centers across the United States between November 19, 2018, and July 14, 2020 (NCT03736928). The study complied with the principles of the Declaration of Helsinki (1964) and subsequent amendments and the International Council for Harmonization of Technical Requirements for Pharmaceuticals for Human Use Good Clinical Practice (GCP). Subjects provided written informed consent, and ethics approval was obtained from each relevant institutional review board (IRB).

The study included adults (aged 18–65 years) with moderate to severe glabellar lines, assessed at maximum frown and graded using the investigator live assessment (ILA) photographic scale and the subject self‐assessment (SSA) static categorical scale. Both ILA and SSA scales comprised a 4‐point grading system: 0 (none), 1 (mild), 2 (moderate), and 3 (severe) (Table 1). Exclusion criteria comprised prior botulinum toxin facial treatment (within 9 months), history of facial surgery or aesthetic procedures, or known allergy/sensitivity to any study product component or to cow's milk protein. Women who were pregnant, planning a pregnancy, or breastfeeding could not enroll. Other exclusion criteria included previous/current eyelid or eyebrow ptosis, amblyopia, cancerous or pre‐cancerous lesions or radiation in the glabellar region or facial nerve palsy, and evidence of inflammation, active infection, or skin disorder (e.g., rosacea) near or in the glabellar region.

**TABLE 1:** Summary of assessment scales

ILA photographic scale	SSA static categorical scale	GAIS grading	FACE‐Q appraisal scales	
			Lines between the eyebrows (bothersome level)	Psychological function (response scale)
0 (none)	0 (none)	Very much improved	1 (not at all)	1 (definitely disagree)
1 (mild)	1 (mild)	Much improved	2 (a little)	2 (somewhat disagree)
2 (moderate)	2 (moderate)	Improved	3 (moderately)	3 (somewhat agree)
3 (severe)	3 (severe)	No change	4 (extremely)	4 (definitely agree)
		Worse		
		Much worse		
		Very much worse		

Note

For FACE‐Q assessments, subjects indicated the extent to which they were bothered by lines between the eyebrows and the degree to which they agreed with a series of statements relating to psychological well‐being.

Abbreviations: GAIS, global aesthetic improvement scale; ILA, investigator live assessment;SSA, subject self‐assessment.

Study vials containing ABO 300 U or placebo lyophilized powder were reconstituted using 1.5, 1.0, 0.75, and 0.60 ml preservative‐free NaCl 0.9% for injection, providing respective treatment doses: 50 U (10 U/0.05 ml injection), 75 U (15 U/0.05 ml injection), 100 U (20 U/0.05 ml injection), and 125 U (25 U/0.05 ml injection). Subjects were randomized (4:1) at baseline (Day 0), to receive either ABO or placebo via 0.05 ml injection at 5 pre‐specified sites in the glabellar region; 2 in each corrugator muscle and 1 in the procerus muscle. Subjects were recruited in a stepwise fashion for the two highest doses. Assessments were conducted post‐treatment at Day 2, Week 1, and Week 2, and then, every 4 weeks from Week 4 through Week 36.

### 2.2 Subject‐reported endpoints

The primary endpoint was composite ≥2‐grade responder rate at Week 4 in those achieving a severity score of 0 (none) or 1 (mild) at maximum frown, evaluated using concurrent ILA and SSA scales (reported in Joseph et al).^21^ The current paper reports subject assessment of efficacy and treatment satisfaction endpoints. A summary of the assessment scales used during the study is shown in Table 1.

Subject assessment of efficacy was conducted at each study visit. SSA responder rate was defined as those achieving ≥1‐grade improvement in glabellar line severity from baseline at maximum frown using the SSA scale. Global aesthetic improvement was defined as those reporting their appearance to be improved, much improved, or very much improved using the 7‐grade scale global aesthetic improvement scale (GAIS). Participants indicated their perceptions of treatment outcome and related QoL impact using the subject satisfaction questionnaire.

FACE‐Q appraisals were conducted at Weeks 4, 12, 24, and 36. Subjects indicated the extent to which they were bothered by glabellar lines (described as lines between the eyebrows) and their degree of agreement with statements relating to psychological well‐being and confidence. For each parameter, the sum of the item scores was converted to a Rasch‐transformed total score according to the FACE‐Q manual.^22^ A higher overall score indicated greater satisfaction. Participants indicated their perceived age (years) based on appearance, relative to their actual age, using a visual analog scale (VAS) score.^23^

### 2.3 Safety endpoints

Safety assessments included the reporting of treatment‐emergent adverse events (TEAEs) and testing for the presence of neutralizing antibodies against ABO using blood samples taken at baseline (prior to treatment) and at Week 36 or in cases of early termination from the study.

### 2.4 Statistical analysis

All statistical analyses presented were planned in the study protocol and performed using the SAS^®^ system (Version 9.4). Sample size calculations showed that inclusion of 80 subjects in each ABO dose group and ≥40 in the placebo group would provide ≥99% power to detect a difference in the composite responder rate between an ABO dose group and placebo. Confidence intervals (CI) were 2‐tailed and constructed at a confidence level of 95%. Efficacy and safety variables were analyzed using the intent‐to‐treat (ITT) population, defined as all subjects randomized and treated with study product. SSA responder rates were evaluated by comparing rates with each ABO dose versus placebo using Fisher's exact tests at a 5% significance level (2‐sided). The study was not powered to compare outcomes between ABO doses, and no correction for multiplicity was used.

## 3 RESULTS

As reported previously, the analysis included 399 subjects receiving either ABO treatment or placebo. Most subjects were female (88.2%) and white (87.2%), and mean age was 48.4 (range: 22–65) years. Severe glabellar lines were reported in 67.9% and 71.7% of subjects when assessed according to the ILA photographic scale and SSA categorical scale, respectively.^21^

Table 2 provides a summary of subject‐reported outcomes (SSA, GAIS, and FACE‐Q), where reported, at baseline, Day 2, Week 4, Week 24, and Week 36 for the placebo group and each of ABO treatment groups. SSA and GAIS responder rates and FACE‐Q appraisal scores were typically improved with ABO treatment, regardless of the dose used.

**TABLE 2:** Summary of SSA, GAIS, and FACE‐Q assessment data (ITT population)

Study visit/treatment group	SSA ≥1‐grade improvement responder rate			GAIS responder rate		FACE‐Q appraisal scales (change from baseline)			
	N	%	*p* value	N	%	Lines between the eyebrows		Psychological function	
						N	Mean change	N	Mean change
Day 2									
Placebo	78	11.5		72	19.4	–	–	–	–
ABO 50 U	80	65.0	<0.001	76	72.4	–	–	–	–
ABO 75 U	80	61.3	<0.001	75	78.7	–	–	–	–
ABO 100 U	80	72.5	<0.001	78	79.5	–	–	–	–
ABO 125 U	81	81.5	<0.001	79	79.7	–	–	–	–
Week 4									
Placebo	78	17.9		74	18.9	74	6.9	74	2.9
ABO 50 U	80	96.3	<0.001	78	98.7	78	55.6	78	17.1
ABO 75 U	80	96.3	<0.001	78	100.0	78	59.4	78	16.4
ABO 100 U	80	97.5	<0.001	78	100.0	78	58.3	78	17.5
ABO 125 U	81	100.0	<0.001	81	100.0	81	59.6	81	14.9
Week 24									
Placebo	78	12.8		68	5.9	68	1.1	68	2.3
ABO 50 U	80	65.0	<0.001	76	64.5	76	25.7	76	11.6
ABO 75 U	80	65.0	<0.001	76	75.0	76	32.1	76	12.0
ABO 100 U	80	60.0	<0.001	72	68.1	72	27.6	72	16.2
ABO 125 U	81	67.9	<0.001	73	82.2	73	40.3	73	9.3
Week 36									
Placebo	78	9.0		67	6.0	67	0.6	67	3.7
ABO 50 U	80	33.8	<0.001	73	27.4	73	15.6	73	9.0
ABO 75 U	80	31.3	<0.001	78	41.0	78	18.6	78	10.6
ABO 100 U	80	38.8	<0.001	75	32.0	75	17.3	75	10.5
ABO 125 U	81	44.4	<0.001	76	48.7	76	24.8	76	10.3

Note

The SSA responder rate reported the proportion of subjects achieving ≥1‐grade improvement in glabellar line severity from baseline at maximum frown using the SSA 4‐point static categorical scale. GAIS responder rate data showed the proportion of subjects in each treatment group reporting improved, much improved or very much improved aesthetic outcomes using the GAIS grading scale. For FACE‐Q assessments, subjects indicated the extent to which they were bothered by lines between the eyebrows and the degree to which they agreed with a series of statements relating to psychological well‐being using a 4‐point scale in each case. The change from baseline is presented for FACE‐Q appraisals. Higher scores reflect improved outcomes. Responder rate *P* values were calculated for ABO treatment groups, versus placebo, using Fisher's Exact Test. N indicates the number of subjects for which data were reported at the study visit and/or the population used to calculate the responder rate or mean change from baseline.

Abbreviations: ABO, AbobotulinumtoxinA; GAIS, global aesthetic improvement scale; ITT, intention to treat; SSA, subject self‐assessment.

**FIGURE 1:**
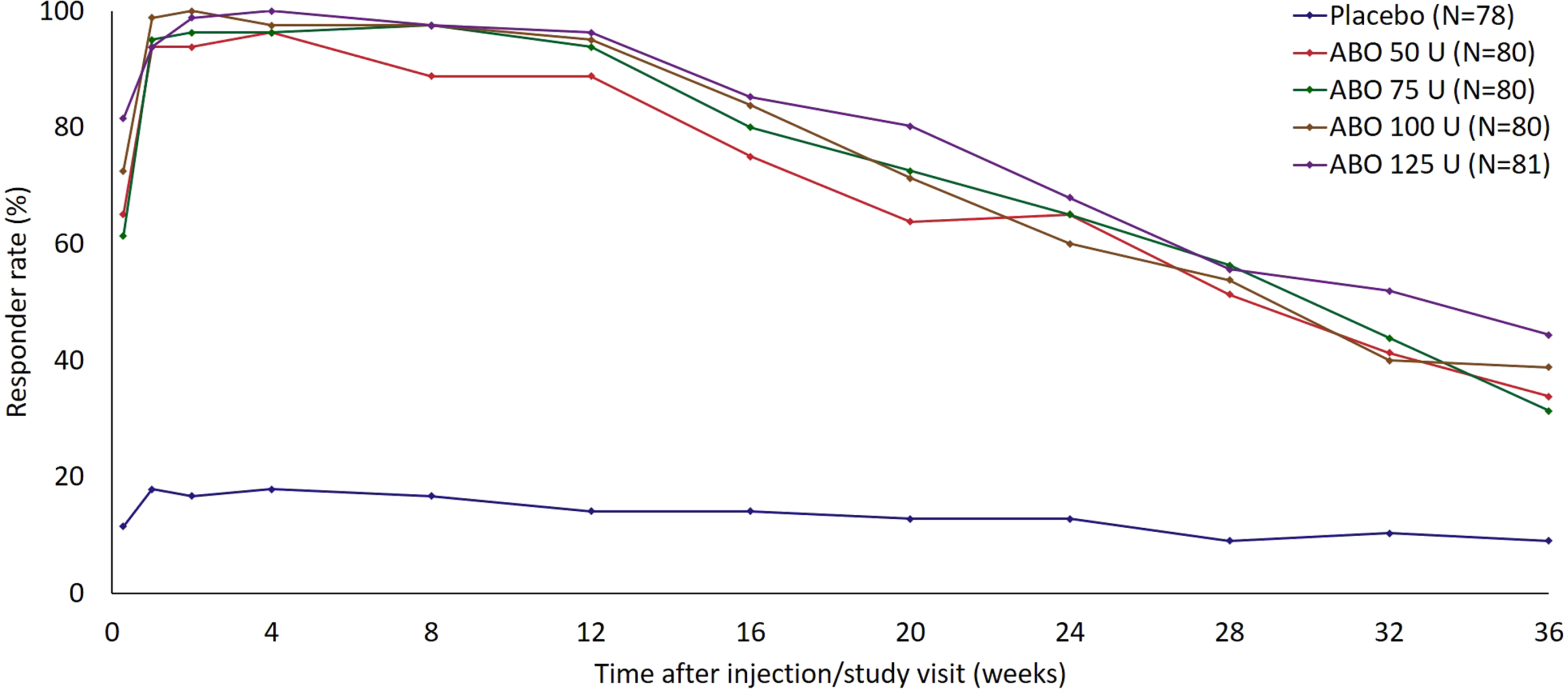
Responder rate among subjects achieving ≥1‐grade improvement in glabellar line severity score at maximum frown assessed using the SSA 4‐point categorical scale (ITT population). A responder was defined as a subject who achieved an improvement in glabellar line severity score of ≥1‐grade from baseline at maximum frown on the SSA scale. Post‐treatment study visits and assessments were conducted on Day 2, Week 1, Week 2, Week 4, Week 8, Week 12, Week 16, Week 20, Week 24, Week 28, Week 32, and Week 36. Abbreviations: ABO, AbobotulinumtoxinA; ITT, intention to treat; SSA, subject self‐assessment

Figure 1 shows the SSA responder rate at each study visit for subjects achieving ≥1‐grade improvement in glabellar line severity from baseline at maximum frown. Responder rate was significantly higher with each ABO dose, compared with placebo, at all study visits from Day 2 through Week 36 (*p *< 0.001). Responder rate was greater with each increased ABO dose, although the study was not powered to evaluate differences between ABO treatment groups. More than 60% of subjects in each ABO treatment group reported ≥1‐grade improvement on Day 2: 65.0% (50 U), 61.3% (75 U), 72.5% (100 U), and 81.5% (125 U), versus 11.5% with placebo. At Week 4, ≥96% of subjects were responders in all ABO dose groups, versus 17.9% with placebo. This effect was maintained among >60% of ABO‐treated subjects to Week 24 and in >31% of ABO recipients to Week 36.

**FIGURE 2:**
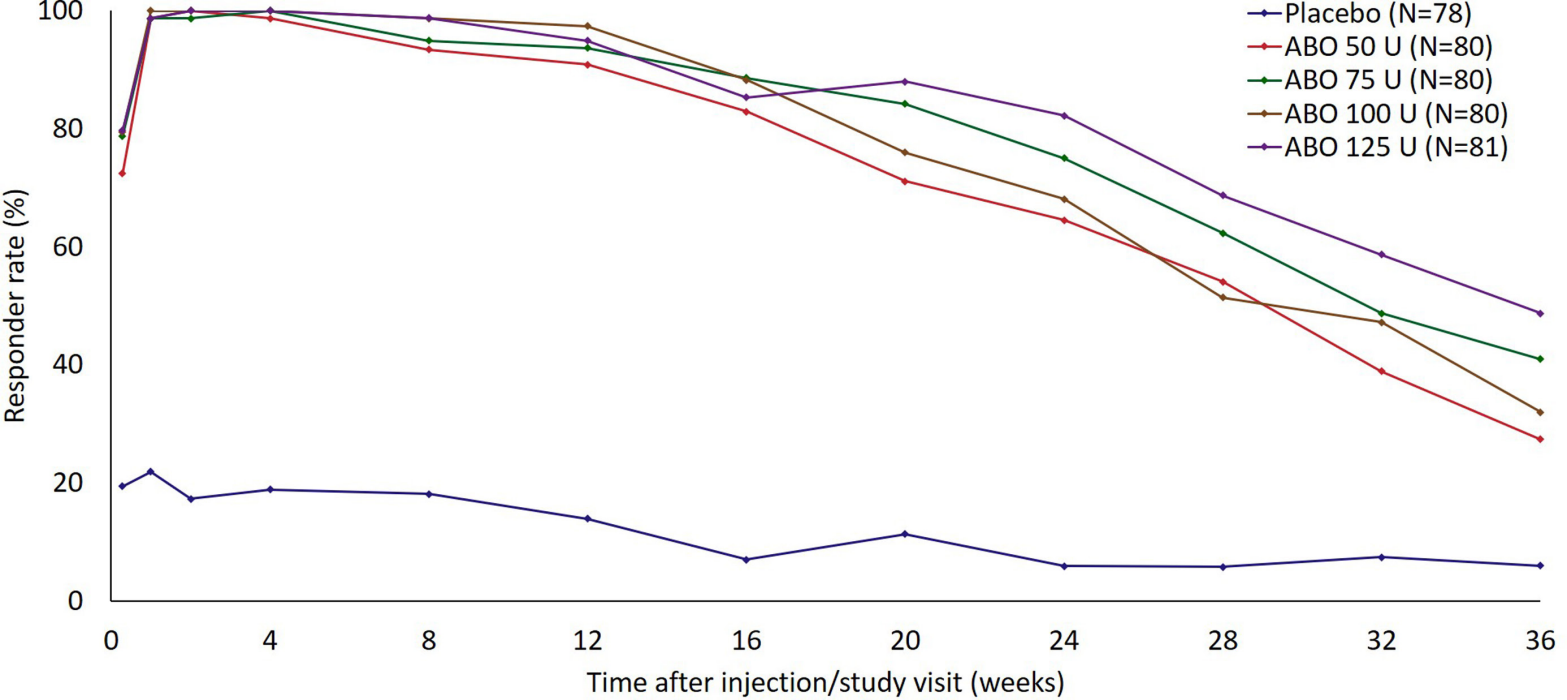
Responder rate at maximum frown assessed using the 7‐point GAIS scale (ITT population). A responder was defined as a subject who indicated that aesthetic appearance was improved, much improved or very much improved from baseline at maximum frown on the GAIS scale. Post‐treatment study visits and assessments were conducted on Day 2, Week 1, Week 2, Week 4, Week 8, Week 12, Week 16, Week 20, Week 24, Week 28, Week 32, and Week 36. Abbreviations: ABO, AbobotulinumtoxinA; ITT, intention to treat; GAIS, global assessment of aesthetic improvement scale

GAIS responder rate data showed the proportion of subjects in each treatment group reporting improved, much improved, or very much improved aesthetic outcomes (Figure 2). All ABO doses were associated with higher responder rates, versus placebo, from Day 2 through Week 36. With the exception of Weeks 12 and 16, the highest responder rates at each study visit were achieved in the ABO 125 U group. More than 72% of ABO‐treated subjects were GAIS responders at Day 2: 72.4% (50 U), 78.7% (75 U), 79.5% (100 U), and 79.7% (125 U), versus 19.4% with placebo. At Week 4, the responder rate was 98.7% with the ABO 50 U dose and 100% for all other ABO doses, while 18.9% were reported to be responders with placebo. Week 24 responder rates were >64% in ABO‐treated subjects, versus 5.9% in the placebo group. Week 36 responder rates were >27% with ABO 50 U and 100 U treatment, 41.0% with the 75 U dose and 48.7% with the 125 U dose, while 6.0% were responders with placebo.

**FIGURE 3:**
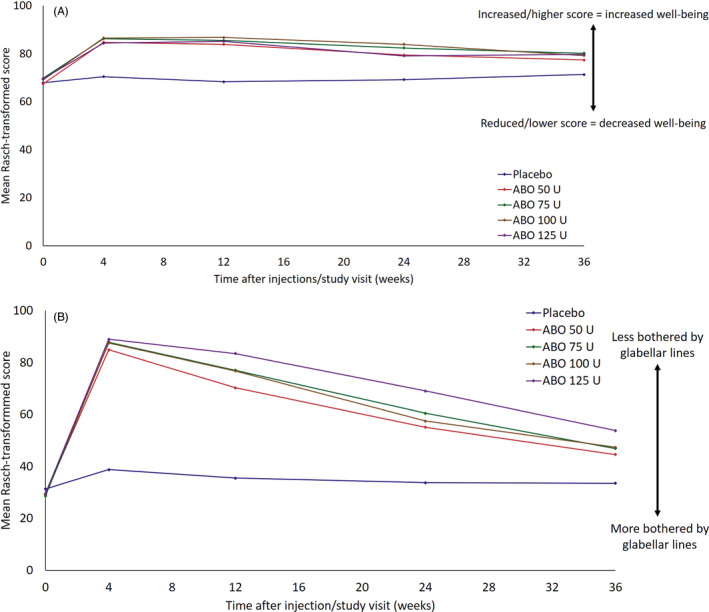
FACE‐Q appraisal mean Rasch‐transformed score, by study visit (ITT population) (A) Psychological assessment. (B) Lines between the eyebrows. FACE‐Q data were reported at baseline, Week 4, Week12, Week 24, and Week 36. Abbreviations: ABO, AbobotulinumtoxinA; ITT, intention to treat.

Figure 3 shows FACE‐Q Rasch‐transformed total scores for subject assessments of psychological function and glabellar lines. For each parameter, a higher score or relative increase from baseline indicated improvement in psychological well‐being or that the participant was less bothered by the severity of their glabellar lines. Psychological well‐being was reported to be improved in each of the ABO dose groups from Week 4 and was maintained above baseline levels for all ABO‐treated participants through Week 36 (Figure 3A). Mean increase from baseline in Rasch‐transformed score regarding well‐being ranged between 14.9 (125 U) and 17.5 (100 U) at Week 4 with ABO treatment, versus 2.9 with placebo. Change from baseline at Week 24 with ABO treatment was between 9.3 (125 U) and 16.2 (100 U), versus 2.3 with placebo. At Week 36, mean change in Rasch‐transformed score from baseline was between 9.0 (50 U) and 10.6 (75 U) with ABO, versus 3.7 with placebo.

Subjects in all ABO dose groups reported improved satisfaction from baseline with the appearance of their glabellar lines after treatment, with the greatest satisfaction occurring at Week 4 (Figure 3B). Subjects reported that they were less bothered by their glabellar lines throughout the study period, with satisfaction levels remaining above baseline through Week 36. Mean increases from baseline at Week 4 ranged from 55.6 (50 U) to 59.6 (125 U) with ABO treatment, versus 6.9 with placebo. At Week 24, ABO‐treated subjects reported changes from baseline of between 25.7 (50 U) and 45.3 (125 U), compared with 1.1 for placebo. At Week 36, mean change in Rasch‐transformed score from baseline was between 15.6 (50 U) and 24.8 (125 U), versus 0.6 with placebo.

**FIGURE 4:**
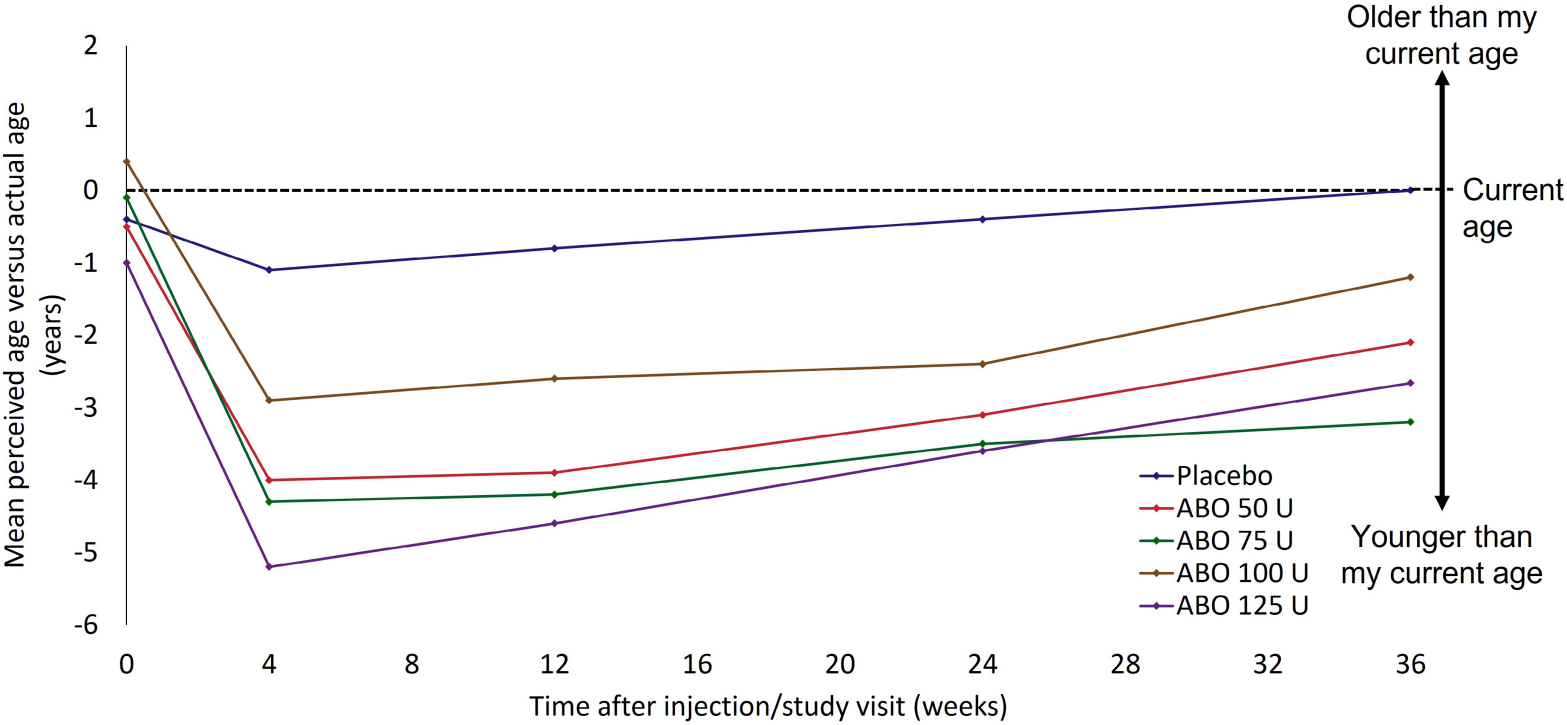
FACE‐Q subject perceived age based on appearance versus their actual age, by study visit (ITT population). FACE‐Q data were reported at baseline, Week 4, Week12, Week 24, and Week 36. Abbreviations: ABO, AbobotulinumtoxinA; ITT, intention to treat

Figure 4 shows FACE‐Q appraisal VAS data for perceived age (years) based on appearance compared with actual age. At baseline, subjects in the ABO 50, 75, and 125 U groups indicated that they looked an average of 0.5, 0.1, and 1.0 years younger than their actual age, respectively. Participants in the ABO 100 U group reported that, on average, they looked 0.4 years older than their real age at baseline. Subjects in the placebo group indicated that they appeared 0.4 years younger than their actual age. ABO‐treated subjects reported themselves to look up to 5.2 years younger (average for the 125 U group) than their actual age from Week 4 through Week 36, while participants in the placebo group indicated that they looked on average ≤1.1 years younger and had returned to looking their actual age by Week 36. Among those treated with ABO, the greatest changes in appearance of age were reported at Week 4 with subjects indicating that they looked between 4.0 (50 U) and 5.2 (125 U) years younger than their actual age. At Week 24, ABO‐treated participants indicated that they looked on average between 2.4 (100 U) and 3.6 (125 U) years younger than their real age. All ABO‐treated groups reported looking on average between 1.2 (100 U) and 3.2 (75 U) years younger their actual age at Week 36.

In the subject questionnaire at Week 4, most subjects (>94%) responded that their appearance looked refreshed after treatment with any ABO dose, and this satisfaction was sustained by >86% at Week 24 and >73% at Week 36. The highest satisfaction levels for refreshed appearance reported throughout the study period were by those treated with the ABO 125 U dose, which remained at or above 90% through Week 24 and at 79% at Week 36. The proportion of ABO‐treated subjects reporting that they felt better/much better following their treatment was >75% in all dose groups at Week 4, and this level of satisfaction was maintained by >61% at Week 24 and >39% at Week 36. The highest levels of satisfaction regarding feeling better/much better at each study visit were reported by those in the ABO 125 U group: 85.2% (Week 4), 82.3% (Week 12), 67.1% (Week 24), and 57.9% (Week 36).

The most popular benefits highlighted by ABO‐treated participants were youthful and less tired appearance. Youthful appearance was reported by >57% of ABO‐treated subjects at Week 4 and this outcome was maintained by >47.4% through Week 36. Once again, those in the ABO 125 U group indicated the highest levels of satisfaction regarding youthful appearance: 70.4% at Week 4 and 67.1% at Week 36. Having a less tired appearance was reported by >69% at Week 4 and >65% at Week 36 in each of the ABO treatment groups. For this parameter, the highest levels of satisfaction were for those treated with the ABO 75 U dose: 82.1% at Week 4 and 70.5% at Week 36. Throughout the 36‐week study period, >89% of subjects in each ABO group said that the treatment results looked natural (range: 89.3% to 100%).

### 3.1 Safety endpoints

In total, 15 non‐serious treatment‐related TEAEs were reported by 13 (4%) ABO‐treated individuals and one (1.3%) placebo recipient. Treatment‐related TEAEs were mild (80%) or moderate (20%) in severity. The most common ABO‐related TEAEs were mild headache (1.2%) and eyelid ptosis (1.2%). All ptosis cases occurred within 16 days of treatment, resolved during the study period (median duration: 75 days) and incidence of ptosis did not increase with higher ABO doses. Mild ptosis was reported by 1 subject treated with ABO 75 U and one receiving ABO 125 U. Two subjects reported moderate ptosis in the 100 U group. No subjects experienced remote spread of toxin effect at any visit. No incidents of seroconversion to ABO neutralizing antibodies occurred during the study period.

## 4 DISCUSSION

Our subject‐reported data showed that individuals with moderate to severe glabellar lines achieved significant improvements in line severity, with high levels of satisfaction and enhanced psychological well‐being following a single ABO treatment, dosed at 50–125 U. Improvements in glabellar line severity of ≥1‐grade were sustained for durations of up to 24–36 weeks (approximately 6–9 months) alongside global aesthetic improvements, with a tendency toward higher responder rates with escalating ABO dose. Participants reported a more youthful and refreshed appearance post‐treatment and indicated that they felt better about themselves. Importantly, >89% of subjects reported a natural look throughout the study at all dose levels (up to the highest dose of 125 U). Safety assessments revealed that all treatment‐related TEAEs were non‐serious and typically mild in intensity. Incidence of ptosis remained low and did not increase at higher ABO doses, no participants experienced remote spread of toxin effect, and no subjects withdrew from the study due to any treatment‐related TEAE. Altogether, these data support the outcomes already reported for the same Phase 2 ABO dose‐escalating study, which demonstrated rapid and high ≥2‐grade composite responder rates, measured concurrently on the ILA and SSA scales, across all ABO doses, with no major differences in safety endpoints compared with the 50 U dose.^21^ The current analysis also provides important insights concerning the psychological benefits that may accompany high levels of satisfaction with successful aesthetic treatments. Satisfaction has become an important indicator of treatment success and is rapidly becoming a standard parameter reported in studies designed to examine the efficacy of aesthetic treatments, such as ABO.^2^, ^5^, ^6^, ^7^, ^10^, ^12^, ^13^, ^14^, ^15^, ^16^, ^17^, ^24^ This reflects the impact of such treatments on the recipient's emotional well‐being and QoL, as well as their decisions regarding further cycles of treatment.^2^, ^5^, ^6^, ^7^, ^10^, ^12^, ^13^, ^14^, ^15^, ^16^, ^17^, ^24^ The data reported herein are therefore essential in understanding the value and influence of ABO dose escalation on recipient perspectives regarding treatment success.

SSA responder rate, for those showing ≥1‐grade improvement from baseline, was 60–68% at Week 24 (approximately 6 months) and 34%–44% at Week 36 (around 9 months) with ABO doses ranging between 50 and 125 U. These SSA data reflect the pattern of improvement observed with investigator assessments for the same study population, as reported by Joseph et al.^21^ ILA assessments showed that up to 69% (range: 52.5%–69.1%) and 31% (range: 17.5%–30.9%) maintained ≥1‐grade improvements at Weeks 24 and 36, respectively, with efficacy and duration of effect tending to be increased alongside the dose.^21^ The subject‐reported GAIS responder rate showed a similar trend of change to SSA and ILA data, with the higher rates tending to be associated with the ABO 125 U dose.^21^ The SSA and GAIS results echo outcomes from other dose‐ranging studies examining treatment efficacy and duration with botulinum toxin type A treatments, while comparing favorably with previous studies examining the efficacy of the licensed ABO and higher dose.^3^, ^4^, ^5^, ^6^, ^7^, ^8^, ^9^, ^10^, ^11^, ^12^, ^13^, ^21^, ^24^, ^25^ Previously published studies comparing dose escalation with various botulinum toxin A products indicated that, compared with the current licensed/approved label dose, an increase in ABO dose of only 2–2.5‐fold extended the duration of treatment effect to approximately 9 months while a 5‐fold increase in incobotulinumtoxinA dose was required to provide the same durability of effect.^5^ A 4‐fold increase in onabotulinumtoxinA dose was associated with the maintenance of aesthetic improvements over a 6‐month period.^5^ Published data also showed that subject satisfaction and perceptions of natural appearance were maintained among those receiving a 2‐fold and 2.5‐fold increase in ABO dose, while the satisfaction with onabotulinumtoxinA treatment decreased when the dose was raised by 3‐fold or more.^5^

Most subjects in the current study (>65%) reported SSA and GAIS improvements from Day 2, regardless of ABO dose. These data are aligned with the ILA responder rates and the median time to onset of treatment effect of 2 days observed in the dose‐ranging study and the literature regarding the timing of aesthetic effect with ABO injections.^3^, ^4^, ^5^, ^6^, ^7^, ^8^, ^9^, ^10^, ^11^, ^12^, ^13^, ^21^

Subject satisfaction and FACE‐Q assessments regarding line severity tended to mirror SSA and GAIS outcomes over time, with higher scores typically being associated with increasing ABO dose. SSA, GAIS, and FACE‐Q assessments revealed that self‐reported improvement in glabellar line severity peaked around Week 4, with higher responder rate and levels of satisfaction remaining well above placebo through 24–36 weeks (approximately 6–9 months) in all ABO treatment groups. Up to two‐thirds of ABO‐treated subjects (61%–67%) were more satisfied with how they felt about themselves at all post‐baseline time points through Week 24 and 39–57% through Week 36. Improvements in satisfaction and psychological well‐being were likely driven by perceptions of more youthful appearance, which were also sustained by up to two‐thirds of ABO‐treated subjects through Week 24 (53–67%) and Week 36 (47%–67%). Subjects reported looking up to 3.6 years younger than their actual age at Week 24 and between 1.2 and 3.2 years younger at Week 36. These results add to the wealth of existing evidence regarding enhancements in psychological functioning and perceptions of age with ABO treatments.^11^, ^12^, ^13^, ^14^, ^24^ Although the study was limited to a 36‐week period, the results show that dose escalation may be associated with longer duration of treatment effect and satisfaction, and these aspects of treatment may be worthy of further investigation in future studies examining the higher ABO doses over an extended follow‐up. The duration of perceived psychological and aesthetic enhancement demonstrated in our study extends beyond the 4–5 months that is generally accepted for ABO treatment effect with the licensed dose, and satisfaction was sustained in many cases for longer than the 5–6‐month period suggested in the APPEAL study.^12^, ^13^ Subjects indicated that their psychological well‐being was improved and their aesthetic results appeared natural and refreshed with all ABO doses throughout the study period. When considered alongside the efficacy and safety evidence already published by Joseph et al, these data provide additional justification for further investigations exploring the influence of variable ABO dosing in the correction of moderate to severe glabellar lines.^21^ Future research in this area would benefit from the examination of a wider and more heterogeneous study cohort to understand whether the treatment outcomes observed in the current study are applicable to a broader and more diverse population.

## 5 CONCLUSION

A single ABO treatment provided significant and sustained improvements in subject‐assessed glabellar line severity over durations of 6–9 months, versus placebo, at doses of 50–125 U. Self‐assessment of effectiveness and duration of treatment effect and GAIS outcomes tended to be improved at higher ABO doses. Importantly, satisfaction levels were high for all ABO doses, with participants reporting a natural, refreshed and more youthful appearance, and improvements in psychological well‐being that extended up to 24–36 weeks (approximately 6–9 months).

## CONFLICT OF INTEREST

Dr. Dayan is a consultant and investigator for Allergan, Galderma, Merz and Revance. Dr. Joseph is an investigator and paid speaker for Galderma. Dr. Moradi is a paid consultant and clinical trial investigator for Galderma. Dr. Lorenc is an investigator for Galderma. Dr. Coleman is an investigator for Galderma. Dr. Ablon is an investigator for Galderma. Dr. Kaufman‐Janette is a paid advisory board consultant and clinical trial investigator for Galderma. Dr. Cox is an investigator and paid advisory board member for Galderma. Dr. Campbell is an investigator for Galderma. Dr. Munavalli is an investigator for Galderma. Dr. Prygova is an employee of Galderma.

## AUTHOR CONTRIBUTIONS

All listed authors have made substantial contributions to the study conception or design, acquisition of data or analysis, and interpretation. They have been involved in drafting the manuscript or revising it critically. Each author has given their final approval of the version to be published and take responsibility for the content, accuracy, and integrity of the work.

## ETHICAL APPROVAL

The authors confirm that the ethical policies of the journal, as noted on the journal's author guidelines page, have been adhered to and the appropriate ethical review committee approval has been received. The US National Research Council's guidelines for the Care and Use of Laboratory Animals were followed. This study was conducted in accordance with the protocol, clinical trial agreement, the ethical principles that have their origin in the Declaration of Helsinki (1964) and subsequent amendments, and the International Council for Harmonisation of Technical Requirements for Pharmaceuticals for Human Use Good Clinical Practice (GCP), and in compliance with applicable regulatory requirements. Institutional Review Board (IRB) approvals: IRB ID: 7008. The study protocol was approved by Sterling IRB (Protocol Number: 43USD1801). The study was implemented in accordance with the requirements stipulated by Sterling IRB, who also conducted continuing review of the study. The date of IRB approval for each study site is listed below, along with the Principal Investigator at each study center.

**Table:** 

Sterling Independent Review Board Chairman: Steven L. Saltzman, MD	
Principal Investigator and study site IRB	IRB approval date
Glynis Ablon, MD Ablon Skin Institute and Research Center, Manhattan Beach, CA 90266	October 19, 2018
Andrew Campbell, MD ETHIQ2 Research housed under Quintessa Aesthetic Center, Mount Pleasant, WI 53403	October 11, 2018
Kyle Coleman, MD Etre Cosmetic Dermatology, New Orleans, LA 70130	October 11, 2018
Sue Ellen Cox, MD Aesthetic Solutions, PA, Chapel Hill, NC 27517	October 22, 2018
Steven Dayan, MD DeNova Research, Chicago, IL 60611	October 19, 2018
John Joseph, MD Clinical Testing of Beverly Hills, Encino, CA 91436	October 23, 2018
Joely Kaufman‐Janette, MD Skin Research Institute, Coral Gables, FL 33146	October 19, 2018
Paul Lorenc, MD Lorenc Aesthetic Plastic Surgery Center, New York, NY 10028	October 17, 2018
Amir Moradi, MD Moradi MD, Vista, CA 92083	October 16, 2018
Girish Munavalli, MD Dermatology, Laser and Vein Specialists of the Carolinas, Charlotte, NC 28207	October 26, 2018

## Data Availability

The data that support the findings of this study are available on request from the corresponding author. The data are not publicly available due to privacy or ethical restrictions.
